# Genomic Architecture of Nestmate Recognition Cues in the Desert Ant

**DOI:** 10.1002/ece3.72726

**Published:** 2025-12-23

**Authors:** Pnina Cohen, Shani Inbar, Eyal Privman

**Affiliations:** ^1^ Department of Evolutionary and Environmental Biology, Institute of Evolution University of Haifa Haifa Israel

## Abstract

Nestmate recognition is the basis for cooperation within social insect colonies. Quantitative variation in cuticular hydrocarbons (CHCs) is used for nestmate recognition in ants and other social insects. To infer genetic effects on CHC variation, we tested for association between CHCs and polymorphic genetic loci to identify potential quantitative trait loci (QTL). We carried out a genome wide association study (GWAS) of CHCs in the desert ant *Cataglyphis niger* by sampling 47 colonies, fully sequencing six workers from each colony, and measuring the relative amounts of their CHCs. Under the Gestalt colony odor model, social interactions between nestmates, in which CHCs are transferred and mixed, are essential in creating a uniform colony CHC profile. Therefore, we carried out a second GWAS between the colonies and their Gestalt odor by averaging nestmate genotypes and comparing them to their averaged CHC amounts. Together, the two analyses identified 99 candidate QTLs associated with 18 out of 34 CHCs. Thirteen clusters of two to four QTLs located within 10 cM of each other were identified, seven of which contained QTLs from both analyses. We conclude that nestmate recognition cues are complex quantitative colony‐level traits with a significant genetic component to their phenotypic variation and a highly polygenic architecture.

## Graphical abstract

This study reveals for the first time the genomic basis for the chemical cues used by social insects for nestmate recognition. We conducted a GWAS to identify QTLs that explain quantitative variation in cuticular hydrocarbons among colonies from a population of the desert ant *Cataglyphis niger* .

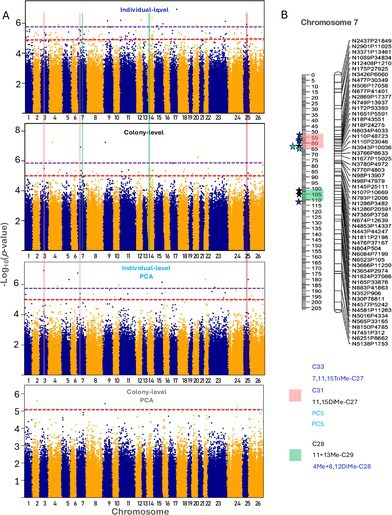

## 1 Introduction

Colonial identity is the foundation and a prerequisite for any social interaction in insect societies. The ability to recognize nestmates and to discriminate them from aliens is needed for protecting the colony's food reserves and brood from being plundered and for preserving territorial control over resources around the nest. In most social insect species, nestmates have high genetic relatedness, in keeping with Hamilton's principles of kin selection and inclusive fitness (Hamilton 1964, 1987), and nestmate recognition is equivalent to kin recognition. A major class of chemical cues that facilitate nestmate recognition are lipids found on the cuticle, mainly n‐alkanes, methyl‐branched, and unsaturated hydrocarbons (Errard and Hefetz 1997; Vernier et al. 2019; van Zweden and D'Ettorre 2010; Lahav et al. 1999; Dani et al. 2005; Hefetz 2007). Insect cuticular hydrocarbons (CHCs) chains vary in length (up to 60, but mostly between 20 and 40 carbons), number and positions of methyl branches, and number and positions of double bonds (Blomquist and Bagnères 2010). Social insect species generally display relatively large numbers of different CHCs (Martin and Drijfhout 2009). While different insect species have qualitatively distinct mixtures of CHCs, intraspecific variation of CHC mixtures is chiefly quantitative. The relative amounts of CHCs are the chemical profile that makes up its identity (Howard 1982; Howard and Blomquist 2005; Blomquist et al. 2018). In ants, the numbers of different CHCs are especially large, even in comparison to other social insects (Martin and Drijfhout 2009), with dozens of distinct CHCs on the body surface of a single individual.

Intra‐colony CHC composition variability is smaller than inter‐colony variability (Lahav et al. 1999; Wagner et al. 2000), and some studies reported that chemical distances (dissimilarity) between CHC profiles of colonies correlate with aggression levels between members of these colonies (Drescher et al. 2010; Foitzik et al. 2007; Blight et al. 2012). Diet, nest material, and other environmental factors were shown to influence ant CHC profiles (M. W. Crosland 1989; Sorvari et al. 2008; Obin 1986) and thereby contribute to nestmate recognition and inter‐colony aggression. Evidence from various studies indicates that genetic factors are also important contributors to the colony‐specific composition of CHCs (reviewed by Sprenger and Menzel 2020). Correlation between genetic and chemical distances was reported for the yellow crazy ant (Drescher et al. 2010), the Argentine ant (Blight et al. 2012), and the clonal raider ant (Teseo et al. 2014). Provost (1991) conducted controlled crosses of *Leptothorax lichtensteini* to produce unfamiliar ants with varying degrees of genetic relatedness, demonstrating aggression is correlated with genetic distance. A genetic component to nestmate recognition is also supported by cross‐fostering experiments in *Formica rufibarbis* (Van Zweden et al. 2010). While these studies provided support for the hypothesis that CHCs are affected by genetic polymorphism, so far no study has identified specific genomic loci that may be responsible for such genetic effects. Conversely, research in *Drosophila* and other model insect species identified a conserved pathway of enzymes responsible for the biosynthesis of CHCs from long‐chain fatty acids (including fatty‐acid synthases, elongases, and desaturases) in oenocyte cells (van Zweden and D'Ettorre 2010) and mechanisms for their trafficking and secretion to the cuticle (that may involve other organs such as the postpharyngeal gland; Bagnères and Morgan 1991).

Two models were suggested for the mechanism that creates a colony specific CHC profile (Crozier and Dix 1979). In the individualistic model, all CHCs found on an individual are self‐produced. Under this model, the similarities between nestmate CHC profiles may be explained by shared genetics between the closely related colony members and by their shared environment. The alternative model is that of the colony Gestalt odor, in which CHCs are transferred and mixed between colony members via allogrooming and/or trophallaxis (transfer of food) (van Zweden and D'Ettorre 2010; Van Zweden et al. 2010; Soroker et al. 1994; Beye et al. 1997, 1998). This serves to reduce intra‐colony variability, which may prevent nepotism in colonies with some kin structure (e.g., in patrilines of a monogyne, polyandrous colony). These two alternative hypotheses were examined in multiple studies, some of which support either of the two models. For example, when colonies of *Cataglyphis iberica* ants were divided into two groups their CHC profiles diverged and inter‐group aggression transpired (Dahbi and Lenoir 1998). The CHC profiles converged after the separated groups were reunited, as expected under the Gestalt model. In another study, on *Rhytidoponera confuse* ants, levels of aggression towards nestmates were measured after members of two colonies were caged together (M. W. J. Crosland 1990). This study concluded that the individualistic component is a greater contributor to the colony chemical identity than the homogenizing Gestalt effect. We note that CHCs may also be sourced from the ants' diet or produced by symbionts. In this study we did not investigate these potential effects, so they may be lumped into the environmental noise in both the individualistic and Gestalt models.

The uniform CHC profile of a colony is a colony‐level phenotype, a phenotype which is the combined output (the average or any other function) of the individual phenotypes of nestmates. As with any other phenotype, colony‐level phenotypes may be affected by internal factors such as genetics, epigenetics, body size, age, and health, and external factors such as nest material, diet, gut microbes, predation pressure, seasonality, and weather. Other examples of colony‐level phenotypes in social insects include alternative social structures, such as the monogyne versus polygyne social structures in fire ants (Ross and Keller 1998), collective activities such as foraging and nest construction (Beckers et al. 1993; Sumpter and Beekman 2003), and social immunity, the defensive actions taken against pathogens and parasites (Sumpter and Beekman 2003; Macevicz and Oster 1976; Huang and Wang 2014). In ants and other eusocial insects, a colony is typically made of close relatives that may be regarded as a single colonial organism or a superorganism (Trivers and Hare 1976; Henri and Roughgarden 2010). Therefore, it is useful to consider a colony‐level genotype as a high‐ploidy genotype of a single entity. These collective genotypes may be studied for their association with any collective colony phenotype, in our case, the homogenized CHC profiles.

Here, we carried out the first genome wide association study (GWAS) to search for the genetic basis of among‐colony variation in CHCs. Based on a pilot study on the power of alternative approaches for executing a colony‐level GWAS (Inbar et al. 2020), we sampled 47 colonies of the ant *Cataglyphis niger* and fully sequenced the genomes of six workers from each colony. We tested for association between genomic polymorphism and the quantities of 34 CHCs using two approaches. The first was a standard GWAS of individual genotypes and phenotypes. We then conducted a colony‐level GWAS, formulated based on the Gestalt model. In the second analysis, we tested for associations between the colony‐level genotypes, represented by the colonial allele frequency in each locus, and colony‐level (averaged) CHC profiles. These analyses revealed candidate loci that underlie inter‐colony variation in CHC profiles, which constitute the genomic basis for nestmate recognition cues.

## 2 Materials and Methods

### 2.1 Study Population

This study used a population sample from Betzet beach on the northern Israeli coastline. This same population was previously described as *Cataglyphis drusus* in previous publications, including mitochondrial sequencing and cuticular hydrocarbon profiling (Eyer et al. 2017), but our recent species delimitation study suggested that this is the same species as *Cataglyphis niger* (Andre, 1881; taxonomic serial no. 576596 in https://www.itis.gov/) because these populations are not differentiated by their nuclear genomic DNA (Brodetzki et al. 2019). We note that some populations of *C. niger* include polygyne colonies (headed by multiple egg‐laying queens) (Brodetzki et al. 2019) and that our recent study revealed that this social polymorphism is associated with a supergene (Lajmi et al. 2024). However, the study population used here is purely monogyne (each colony is headed by a single queen) (Eyer et al. 2017; Brodetzki et al. 2019). The supergene is always homozygous (MM) in monogyne samples (and always heterozygous MP in polygyne samples), so we do not expect it to affect the population genetics and the performance of GWAS in this study population. *C. niger* queens are mated with 2–9 males (polyandrous), resulting in an average of 0.26 within‐colony genetic relatedness (Eyer et al. 2017; Brodetzki et al. 2019). Colonies of *C. niger* and other closely related species are often polydomous, with multiple neighboring nests housing the members of a colony (Leniaud et al. 2011; Dillier and Wehner 2004). They are scavengers that primarily forage for dead insects (Boulay et al. 2017). *C. niger* is widespread across Israel, especially common in sandy dunes and arid regions (Boulay et al. 2017).

### 2.2 Sampling Scheme

**FIGURE 1:**
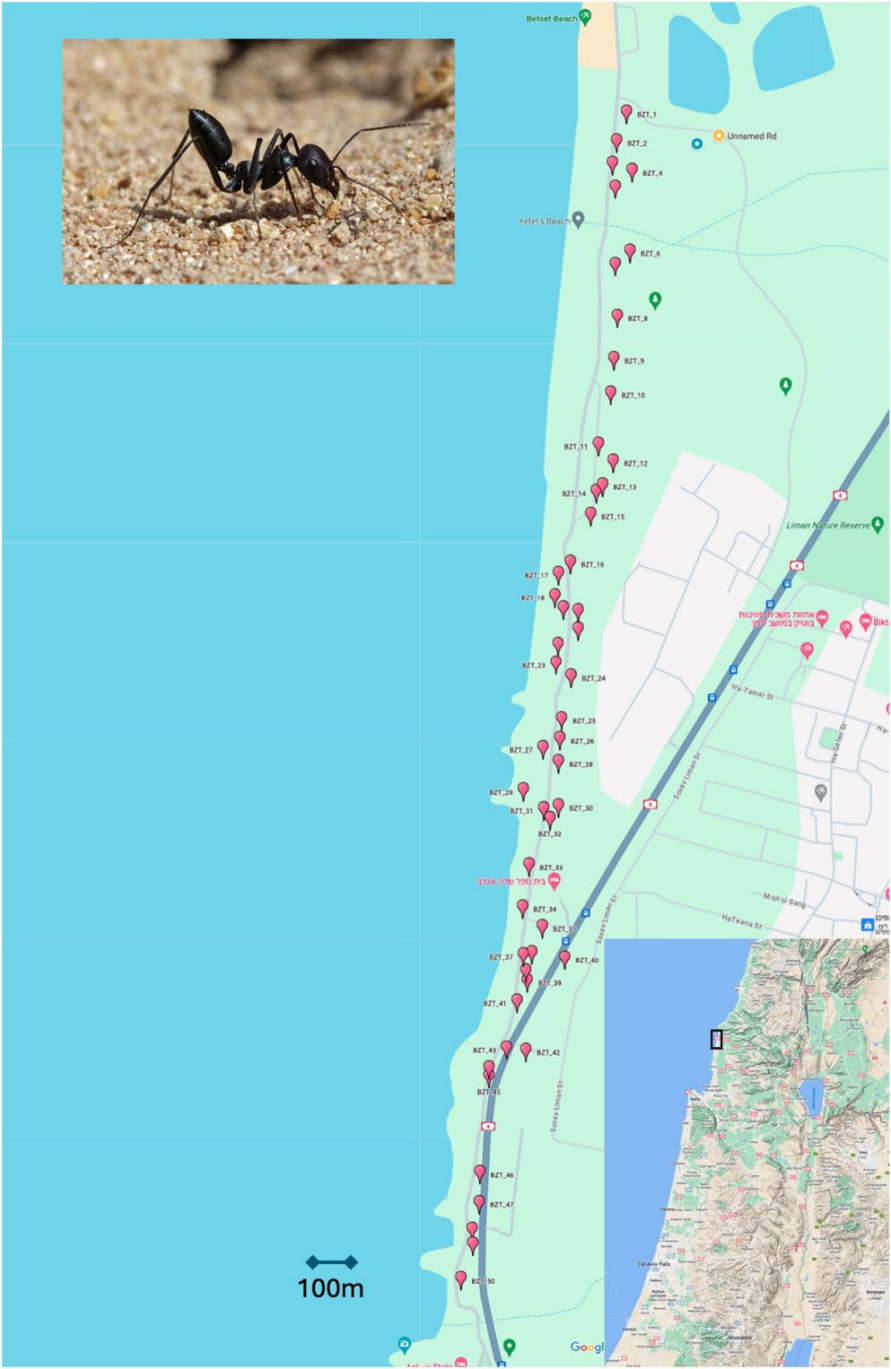
Map of 50 sampled *Cataglyphis niger* nests along a transect in Betzet Beach in northern Israel (from 33.07868 N 35.10705 E to 33.05162 N 35.10245 E).

We collected ants from nests dug along a 4 km transect in Betzet beach (from 33.07868 N, 35.10705 E to 33.05162 N, 35.10245 E; Figure 1) between April 24 and May 10, 2016. Ants were collected from each nest to a plastic box, brought back to the lab alive, and then frozen in a −80°C freezer on the same day. At least 30 workers were randomly sampled from each of 50 nests, 47 of which were used in this study (35.10297 N, 33.05339 E–33.05162 N, 35.10245 E). The other three nests were used in the pilot stage of this GWAS (Inbar et al. 2020).

### 2.3 Behavioral Assays

There exists extensive literature on nestmate recognition studies in *C. niger* showing that CHCs are a major component of the chemical cues that workers use to discriminate non‐nestmates from nestmates (Lahav et al. 1999; Soroker et al. 1994). Behavioral assays were previously conducted in our study population, demonstrating that workers from different colonies discriminate nestmates from non‐nestmates even between nests that are only 100 m apart (Brodetzki et al. 2019). We conducted dyadic aggression assays to compare encounters between nestmates and between non‐nestmates. The results confirm that the nests we sampled indeed exhibit the ability for nestmate recognition. See Inbar and Privman (2018) for the details of this behavioral assay.

### 2.4 Pilot Study, Experimental Design, and Power Analysis

Before sequencing and analyzing our GWAS samples we conducted a pilot study to determine which experimental design gives maximal statistical power to detect QTLs for colony‐level traits. In this pilot, we compared different combinations of population genomic methodologies and approaches, including whole‐genome versus RAD sequencing and individual versus pool sequencing, and also used empirical results as a basis for a simulation study to assess statistical power. Our simulations included varying degrees of genetic versus environmental effects and the mixing of the individual phenotypes at the colony level. See the full details in Inbar et al. (2020). Based on the conclusions from this study we chose to conduct whole genome sequencing on six individual samples per colony. Naturally, the more samples the better, but the simulations showed diminishing returns for increasing the number of samples per colony. We note that this is true even though our study population consists of multiply mated queens (we simulated up to 10 matings per queen, in line with estimates of 2–9 patrilines per colony in *C. niger*; Brodetzki et al. 2019). Based on these results we expect our GWAS to have a 37% and 64% probability to detect QTLs that explain 23% and 52% of the variation (percent explained variability [PEV]) in the studied trait, respectively, with a *p*‐value of 5% after correction for multiple testing. Since ours is the first CHC GWAS or QTL mapping study in ants, we could not predict the expected PEV for an ant CHC QTL, so we did not know in advance whether 23% is a biologically realistic effect size in our study population.

### 2.5 Whole Genome Sequencing

For six individuals from each of the 47 nests, DNA was extracted from the head and abdomen (QIAGEN AllPrep DNA/RNA Mini kit) and the thorax was used for the chemical analysis of CHCs (see below). A uniquely barcoded genomic library was constructed for each sample following the standard protocol of the Illumina TruSeq Nano DNA Library Prep kit. Briefly, a Covaris S220 sonicator was used for DNA shearing aiming for 550 bp inserts, ends were repaired, and the fragments were size‐selected and purified using magnetic beads (supplied in kit). Then, an adenosine was added to the 3′ end of the blunt fragments, individually barcoded adaptors were ligated, and ligated fragments were amplified (eight PCR cycles). Libraries were pooled at equal quantities as three separate pools, each of which was sequenced in two lanes of paired‐end 150 bp reads on a HiSeq X Ten Illumina sequencer (six lanes in total).

### 2.6 Sequence Data Processing and Genotype Calling

#### 2.6.1 Raw Reads Pre‐Processing

Whole‐genome sequence data were successfully obtained for 276 of the 282 samples (four from colony 10, five from colonies 18, 19, 22 and 29 and six from each of the other colonies). A total of 5,941,275,998 reads of 150 bp were sequenced, with an average of 21,526,362 reads per sample, which is an average genome coverage of 12× (0.5×–28×). Duplicate read pairs were removed and low‐quality reads and adaptor contaminations were trimmed or removed using Trimmomatic (Bolger et al. 2014) version 0.36, leaving sequences that were at least 75 bp long. The total number of remaining reads was 5,527,107,050 (93% of the raw data). Reads were aligned to the *C. niger* reference genome (version Cnig_gn2; NCBI BioProject accession PRJNA1168125) using Bowtie2 (Langmead et al. 2009) version 2.2.5 run with the “very‐sensitive” and “end‐to‐end” settings. 3,216,552,155 reads (54% of raw reads) remained after filtering out alignments with three or more mismatches or multiple mapping, which were defined as reads with a second best match that has less than twice the number of mismatches as the best match. HaplotypeCaller of the Genome Analysis Toolkit (GATK) package (McKenna et al. 2010) version 3.7 was used to create a single nucleotide polymorphism (SNP) catalog containing 2,792,460 SNPs.

In a first stage of filtering, we removed indels and SNPs with more than two alleles from the catalog, leaving 2,413,918 SNPs. Sixteen samples (all four of colony 10, three of colony 11, two of colony 9, and one of colonies 2, 8, 15, 16, 25, 40, and 42) were removed because they were missing genotype data in more than 50% of the loci, leaving 260 individuals from 46 colonies in the analysis.

#### 2.6.2 SNPs Catalog for GWAS

In a second stage of filtering we required for each locus to have genotype calls for at least 80% of the samples with a minimum number of 6 reads per genotype call, a maximum of 250, and a minimum minor allele frequency (MAF) of 15%. In order to remove false SNPs that are the result of collapsed repetitive sequences in the reference genome assembly, a control for these was made using three haploid male *C. niger* sampled in Betzet Beach (33°4′40.88′′ N/35°6′33.97′′ E) that were fully sequenced with high coverage (177×, 55×, and 72×). This was done by creating a blacklist of loci where one or more of these haploid samples appears to be heterozygous, which indicates an artifact because male ants are haploid. All SNPs within 600 bp of such heterozygous loci were removed from the catalog. Additionally, excessively covered SNPs, defined as loci with an average number of reads per sample > 20 (average genome coverage + 2 × standard deviation—see Figure S1A). We then removed 14 samples that had more than 60% missing genotypes after this filtering, and as a result had to exclude colony 9 because it had only two samples left. We were left with 244 individuals from 45 colonies (three from colony 11 and 29, four from colony 7, 8, 25, and 32, five from colonies 2, 14, 15, 16, 18, 19, 20, 22, 26, 30, 40 and 42 and six from all the rest) and 171,570 SNPs. The average coverage of this filtered SNP catalog across the remaining 244 individuals was 10.2× (calculated by the actual number of reads covering the SNPs). Overall, 6,348,274 (15.2%) of all possible 41,863,080 locus X sample combinations did not have genotype call (missing data).

#### 2.6.3 Colonial Genotype Processing and Calculation

For the colony‐level analysis, we required that every locus had genotype calls in at least three individuals in each colony, further reducing the number of SNPs to 171,355. Overall, 659,721 (8.6%) of possible 7,710,975 locus X colony combinations did not have genotype call (missing data). We defined colony‐level genotypes as the estimated allele frequency of the colony at every locus, which essentially means that a colony is treated as a single polyploid organism. For example, if the genotypes of six members of a colony are 00, 01, 01, 11, 11, 11, where 0 and 1 represent the two alternative alleles in a locus, their individual genotypes were coded in the input file for the GWAS analysis as 0, 1, 1, 2, 2, 2 and their colony‐level genotype was calculated as an average: $\frac{\left(0+1+1+2+2+2\right)}{6}=1\frac{1}{3}$.

### 2.7 Kinship Inference

#### 2.7.1 Individual‐Level Kinship Based on the Whole‐Genome Data

To create the kinship matrix on the individual level, we chose stringent filtering criteria to ensure low missing data rates and allowed low frequency alleles, which provide useful information for inference of identity‐by‐descent (IBD) of haplotypes. We filtered the SNP catalog of the whole‐genome sequenced samples (after first stage filtering) so that each locus had genotype calls in at least 88% of the samples with a minimum of six reads, a maximum of 250, and a minimum MAF of 1%. Here the MAF cutoff was reduced relative to the GWAS analysis above because rare alleles are a powerful signal for kinship. A MAF of 1% still requires a minimum of two samples that share the minor allele (if homozygous; more if heterozygous), which makes it unlikely to be an artifact due to sequencing errors. We controlled for and filtered out reads that were mapped to suspected collapsed repetitive sequences in the reference as described above, and similarly removed 16 samples from the analysis due to insufficient genotype calls. This resulted in a catalog of 88,238 SNPs. Using the program Beagle (Browning et al. 2018), we imputed and phased the catalog to the total of 9634 phased segments. To assess the performance of imputation in our study we masked a subset of the genotypes of a sample and then used Beagle to impute them. The imputation error rates were 7.8%, 9.6%, and 12.3% for imputing 10%, 25%, or 50% of missing genotypes, respectively. The refined‐IBD algorithm (Browning and Browning 2013) was used to identify shared haplotypes between the samples. A kinship matrix was created based on the total length of IBD haplotypes shared between every pair of samples.

#### 2.7.2 Colony‐Level Kinship Based on RAD‐Sequencing

To better estimate kinship between colonies, one individual from each of the 47 colonies was chosen to be sequenced with greater sequencing depth using a reduced‐representation genomic sequencing approach: the double‐digest Restriction‐site Associated DNA sequencing (ddRAD‐seq) protocol. The rationale for this is that kinship inference by detection of haplotypes that are identical by descent (IBD) between pairs of samples depends on low genotype error rates. Using ddRAD‐seq, we could sequence a smaller number of loci to a much greater depth (65×—see below), which reduces the genotype error rate.

#### 2.7.3 Restriction‐Site Associated DNA (RAD) Sequencing

For one worker per nest, a library was constructed according to a ddRAD‐seq protocol based on protocols from Peterson et al. (2012) and Brelsford et al. (2016). Briefly, DNA was digested by two different restriction enzymes (*Eco*RI as the rare‐cutter; *Mse*I as the frequent‐cutter) and ligated to barcoded adaptors for multiplexing. Products were amplified using Q5 Hot Start Polymerase (NEB), with the number of cycles reduced to 20, the number of replicates increased to four, and starting DNA volume increased to 6 μL. Also, primers and dNTPs were added to the final thermal cycle in order to minimize single‐stranded or heteroduplex PCR products. The libraries were sequenced in one lane of paired‐end, 150 bp reads, on a HiSeq X Ten Illumina sequencer.

#### 2.7.4 Pre‐Processing of RAD‐Seq Data

A total of 823,087,220 reads were sequenced from 47 workers (one from each of the colonies), which is an average expected depth of 65× (14×–105×) based on the number of restriction sites in the reference genome. The raw reads were initially processed using the *Stacks* pipeline (Catchen 2013) version 2.2. Low quality reads were discarded if their quality score dropped below an averaged phred score of 10 in a sliding window of 15% of the reads' length. After this initial processing, 736,899,655 reads that were assigned to a sample remain (90% of raw data). The reads were mapped to the reference genome of *C. niger* (version Cnig_gn2; NCBI BioProject accession PRJNA1168125) using *Bowtie2* and filtered using the same criteria as for the whole‐genome data above. After this filtering, 718,157,408 alignments remained. We continued to analyze the mapped sequences using the *gstacks* program of the *Stacks* pipeline and created a catalog containing 90,006 SNPs for the 47 samples.

#### 2.7.5 Combining the Catalog With Additional Samples From Across Israel

To increase the number of samples and thereby improve SNP calling, imputation and phasing, we made use of another data set that included 103 RAD‐sequenced *C. niger* and *C. israelensis* individuals sampled across Israel (described in Brodetzki et al. 2019). The combined catalog that was created from the 150 individuals contained 346,940 SNPs.

The following filtering parameters were used: each locus was required to have a genotype call in at least 90% of the samples, with the minimal number of reads of 3, a maximum of 4000, and had a minimum MAF of 1.5%. We controlled for collapsed repetitive elements by using the blacklist described above and by removing excessively covered SNPs, which were defined as loci with a mean number of reads > 250 (mean coverage + 2 × standard deviation—see Figure S1B). After filtering, 21,179 polymorphic sites remained in the combined catalog.

#### 2.7.6 Colony‐Level Kinship Inference Based on RAD‐Seq Data

As with the whole‐genome data, the RAD‐seq SNP catalog was imputed and phased using Beagle and refined‐IBD to a total of 502 phased segments across the genome. Shared IBD haplotypes between individuals were identified, and a kinship matrix was created based on the total length of IBD haplotypes shared between every pair of samples representing 47 colonies.

### 2.8 Chemical Analysis of Cuticular Hydrocarbons

CHCs were measured for the same six workers from each of the 47 nests that were used for genomic sequencing of DNA extracted from their head and abdomen. Thoraces were individually immersed in hexane to extract non‐polar cuticular lipids from each sample separately. Samples were analyzed after being kept in a −80°C freezer. Initial analysis was conducted by gas chromatography/mass spectrometry (GC/MS), using a VF‐5ms capillary column, temperature‐programmed from 60°C to 300°C (with a 1 min initial hold) at a rate of 10°C per min, with a final hold of 15 min. Compounds were identified according to their fragmentation pattern and respective retention time compared to synthetic standards (linear aliphatic hydrocarbons from C10 to C40 supplied by Supelco), as in previous studies of this and other populations of the species (Brodetzki et al. 2019). See Figure S2 for example chromatogram and spectra of diagnostic ions of identified compounds. Subsequently, all samples were assayed quantitatively by flame ionization gas chromatography (GC/FID), using the above running conditions. Peak integration was performed using the program Varian Galaxie (version 1.9) and relative amounts of each compound were calculated. The relative amounts of each CHC were calculated for each sample (Table S3). We confirmed that different nests have distinct CHC profiles using a linear discriminant analysis (LDA *p*‐value < 0.0001; Figure S3). For the colony‐level analysis, the relative amounts were averaged between the members of each colony.

#### 2.8.1 Normalization of the CHC Relative Amounts

Outliers to the distribution of each CHC in each of the two datasets (individual relative amounts and averaged colony relative amounts) were removed. These were defined as the amounts that were 1.5 times higher or 1.5 times lower than the interquartile range (the difference between the 75th and 25th percentiles of the distribution). For each dataset, we normalized the distribution of the remaining values using the R package *bestNormalize (*Peterson and Cavanaugh 2019), which chooses the best fitting normalization function for the dataset on the basis of the Pearson's *P* statistic (see Table S1 for more details).

### 2.9 CHC GWAS

In order to test for a genetic basis of variation in CHCs, we conducted a genome wide association study (GWAS) over the relative amounts of each CHC in two levels. The first is the individual level, in which we used the catalog of genotypes created for the 244 samples in 45 colonies, as described above, and their relative amounts of CHC. The second level is the colony level, in which we used the catalog of colony‐level genotypes created for the 45 colonies, as described above, and their averaged colony‐level CHC relative amounts.

The standard practice for GWAS is the use of a mixed linear model (MLM), in which the population structure and the genetic variation are represented as fixed effects, and kinship between samples as random effects. Here, for each dataset (individual or colony level), we to accounted for sample relatedness by the respective kinship matrix (whole‐genome or RAD sequencing data for the individual‐ or colony‐level datasets, respectively) and for population structure by a principle component analysis (PCA) of the SNPs over 45 dimensions in the individual‐level analysis and 8 dimensions in the colony‐level analysis. The number of dimensions was chosen based on the fit of quantile–quantile (Q–Q) plots.

To complement the trait‐wise GWAS, we performed GWAS on the principal components (PCs) of the trait matrix to account for shared variation and uncover latent phenotypic structures reflecting shared genetic architecture. The PC‐wise GWAS was conducted over the PCs that explained at least 4% of the variation. In both individual‐ and colony‐levels, these included the first six PCs (see Figure S4).

#### 2.9.1 Execution of GAPIT

Genomic Association and Prediction Integrated Tool (GAPIT) (Lipka et al. 2012) is an R package which executes the Efficient Mixed Model Association (EMMA) (Kang et al. 2010) method, a computationally efficient algorithm for the study of genomic association using MLM. We ran the program for each CHC in each dataset separately using the Multiple Loci Mixed Linear Model (MLMM), which is implemented in GAPIT by a forward‐backward stepwise MLM regression. In each step, the loci that were found to be significantly associated with the trait in the former step are added to the model as cofactors. Accounting for the effect of multiple loci on the trait reduces residual variation in the trait and allows for greater statistical power in QTL detection.

We evaluated how well the model accounts for population structure and kinship using Q–Q plots: plotting the negative logarithm of the assessed *p*‐values and comparing them to the expected distribution of *p*‐values under the null hypothesis of no association, as calculated by GAPIT (see Figures S5 and S6). Of the 171,570/171,355 loci analyzed, 155,815/155,807 were mapped to a linkage group on the genetic map (Peterson et al. 2012).

#### 2.9.2 Empirical Permutation Test

To account for additional unknown confounding factors and other potential problems in the model that might lead to false inference of association, we performed a permutation test to empirically calculate a null distribution of *p*‐values when no association between the genotype and phenotype exists: for each CHC, we randomly shuffled the trait values between the samples. Using the shuffled traits and leaving the other model parameters unchanged, we reran GAPIT 100 times. We did this for both the individual‐level and colony‐level analyses. For each trait, the original *p*‐values of the original run were re‐evaluated by comparing them to the distribution of the *p*‐values in the shuffled datasets. The *p*‐values were further corrected for multiple testing using the Benjamini–Hochberg false discovery rate (FDR) method (Benjamini and Hochberg 1995). We report associations above multiple significance thresholds, with FDR ranging from 5% to 30%. While it is not common to report results with 30% FDR, we find it useful to include this lower threshold. For example, in the individual‐level GWAS we report 69 loci above this threshold. While 30% of them are expected to be false positives, these results show that approximately 48 loci (70%) are associated with CHCs across the genome. We ask our readers to keep this in mind while considering our results.

All scripts used in the bioinformatics pipeline were submitted to the Zenodo repository (https://doi.org/10.5281/zenodo.16729334).

## 3 Results

We conducted a GWAS to identify the genetic basis of variation in CHCs. We tested for associations between the relative amounts of 34 CHCs and genotype calls at 171,570 SNPs identified through the whole‐genome sequencing of 244 workers from 45 colonies of *C. niger* (3–6 ants from each colony). As part of the analysis, relatedness between samples is accounted for by a kinship matrix and population structure is accounted for using a PCA. This analysis was conducted both at the individual level and at the colony level, where the colony phenotype was defined as the averaged CHC level in the colony and the colony genotype as the allele frequency in the colony. Each locus was assigned a *p*‐value for its association with each of the traits, which we corrected for multiple testing into *q*‐values.

### 3.1 Population Structure and Kinship Between Colonies

It is important to account for population structure and kinship between samples before conducting a GWAS. We analyzed population structure using principal component analysis (PCA) of RAD‐seq data from one sample of each of the 47 colonies. This analysis did not show any clear population structure (Figure S7). Kinship among samples was inferred from the whole‐genome sequencing data based on the total length of shared IBD haplotypes (Figure S8A). As expected, and with very few exceptions, stronger kinship was identified between samples collected from the same nest (intra‐colony kinship) relative to kinship between nests (inter‐colony kinship). The inter‐colony kinship between members of colonies 33 and 47 was found to be similar to intra‐colony kinship. A possible explanation is that the queens of these two colonies were closely related, either a mother and a daughter (50% related) or full sisters (75% related). There is also high relatedness between some members of colonies 40, 41 and 42. There appears to be some kinship among colonies 1–20 (northern part of the transect) and among colonies 32–47, in line with the limited structure observed in the PCA results. Another possible explanation for relatedness is that workers may be exchanged between nests. In this case not all nestmates would be sisters, but this is not what we see in the intra‐nest kinship scores. Kinship was also inferred from more deeply sequenced RAD‐seq data from one representative of each colony. High sequencing depth is expected to give lower genotype error rate, which is important for kinship inference. Various levels of inter‐colony kinship were observed, mostly between neighboring nests, which also supports the hypothesis of short‐range dispersal (between colonies 16 and 19; colonies 34 and 37; colonies 35, 40, 41 and 42; and colonies 46 and 47; see Figure S8B).

### 3.2 Individual‐Level GWAS

**FIGURE 2:**
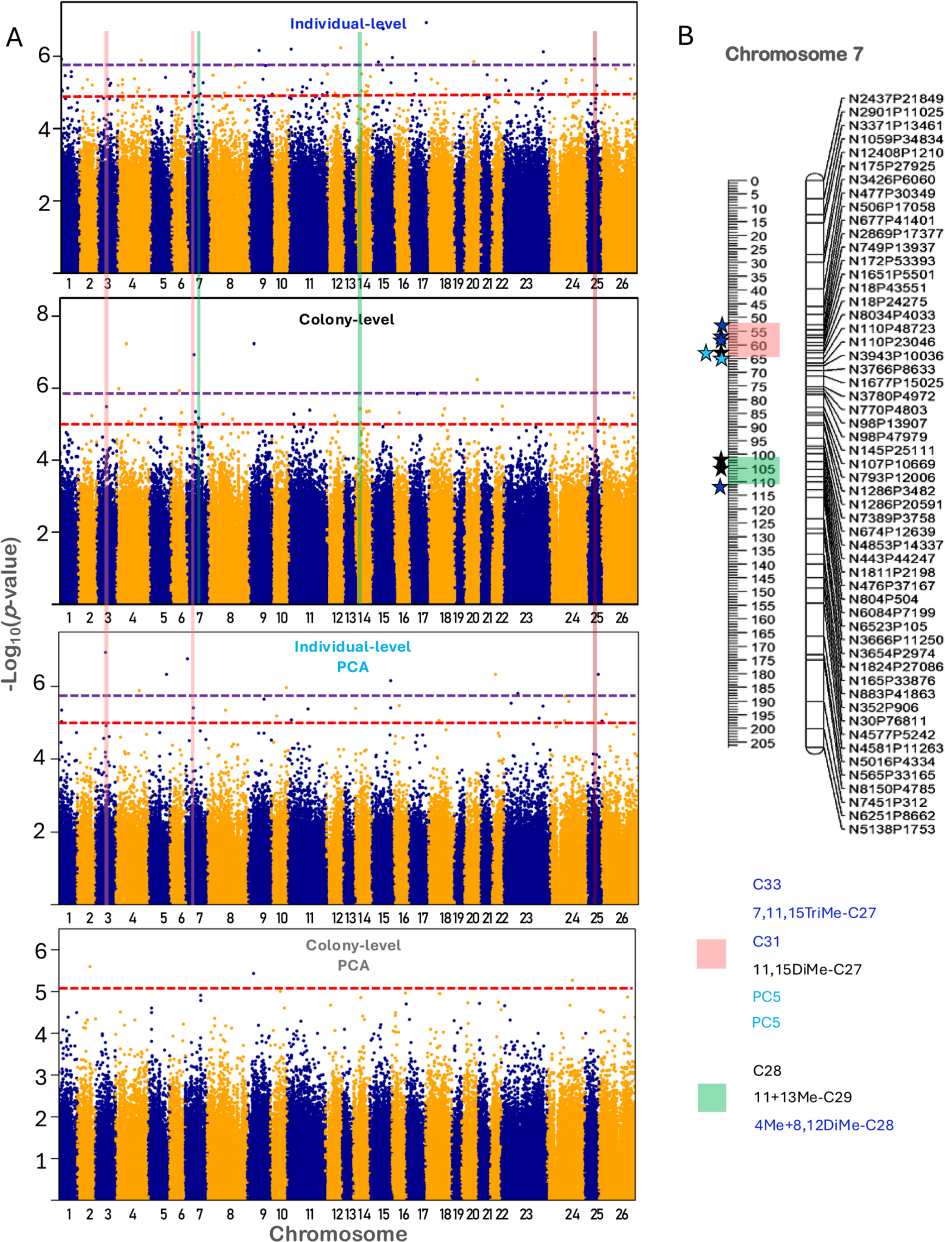
Manhattan plots of associations between loci and CHCs. (A) The top two plots show SNPs associated with CHC traits on the individual‐ and colony‐level, while the bottom two plots show associations with the first six PCs of the CHC profiles. Loci above the purple dotted line are statistically significant with false discovery rate (FDR) of 10%. Loci above the red dotted line are statistically significant with FDR of 30%. Five QTL clusters with at least three QTLs identified in both individual and colony‐level analyses are marked on chromosomes 3, 7, 14, and 25. (B) Two of these clusters on chromosome 7 are presented in detail. Stars mark each significant locus. The associated CHCs are shown in the order of their appearance on the chromosome (trait‐wise individual‐level QTLs in blue and colony‐level QTLs in black, PC‐wise individual‐level in light‐blue).

The individual‐level GWAS identified 13 QTLs that were found to be associated with one of the CHC with the corrected *q*‐value < 0.1, three of which were mapped to genes (Figure 2; Table 1). Two of the QTLs found associated with 5Me‐C25 were only 4.6 cM apart on chromosome 15. Additionally, 3, 13, 39, and 69 loci were found to be associated with one of the traits with a *q*‐value < 5%, 10%, 20%, and 30%, respectively. Because many CHCs may be correlated, we also used PCA to summarize the variation in CHCs and conducted GWAS of the first six PCs. This analysis identified 4, 14, 21, and 32 QTLs with *q*‐value < 5%, 10%, 20%, and 30%. Interestingly, most of these QTLs were associated with PC4 and PC5 (8 and 14, respectively). Of these, 26 QTLs were located within a gene or within 2000 bp upstream from a gene's start codon (gene hits), and four of the genes had a QTL in an exon. We note that for the lower significance threshold, 30% of the associations are expected to be false positives, so approximately 48 out of 69 putative QTLs are expected to be true positives. We also used Q–Q plots to assess the distribution of *p*‐values under the null hypothesis of no association. These plots show that the association test was well‐controlled for potential artifacts such as kinship or non‐normal trait distributions that might result in false positives (Figures S5 and S6).

**TABLE 1:** Candidate loci and genes that were found to be associated with CHCs in the individual‐level GWAS.

Trait	*q*‐value threshold				Genes (*q*‐value)
	0.05	0.1	0.2	0.3	
3Me‐C25	—	—	—	4	Discoidin domain‐containing receptor 2 (0.282)
5Me‐C25	2	4	7	7	Sodium‐coupled monocarboxylate transporter 1 (0.106)
					Protein Wnt‐10a (0.106)
					2‐Oxoglutarate and iron‐dependent oxygenase domain‐containing protein 3 (0.093)
					MIP18 family protein galla‐1 (0.093)
4,12DiMe‐C26	—	—	2	5	Putative sperm flagellar membrane protein (0.280)
11 + 13Me‐C27	—	—	1	1	Putative protein (0.173*)
7,11,15TriMe‐C27	1	1	4	8	DDB1‐ and CUL4‐associated factor 5 (0.018)
					Troponin I (0.265)
12Me‐C28	—	3	3	5	Follistatin (0.094)
4Me + 8,12DiMe‐C28	—	1	4	10	Pyridoxal‐dependent decarboxylase domain‐containing protein 1 (0.211)
					39S ribosomal protein L12, mitochondrial (0.211)
					Putative protein (0.211*)
					Putative protein (0.168*)
C29	—	—	2	5	Protein phosphatase Slingshot homolog 1 (0.285*)
7,11,15TriMe‐C29	—	—	—	3	Putative protein (0.254)
2Me‐C30	—	—	1	4	
4Me‐C30	—	1	1	1	
C31	—	—	8	9	Cadherin‐99C (0.144)
					Kinesin‐like protein KIF6 (0.144)
					Putative protein (0.144*)
					Putative protein (0.226)
C32	—	3	5	5	Putative protein (0.091)
C33	—	—	—	2	Domain of unknown function (0.282)
Total (CHCs)	3	13	39	69	
PC2	0	0	1	4	Putative protein (0.225)
PC3	0	4	4	5	Calcium‐transporting ATPase sarcoplasmic/endoplasmic reticulum type (0.238), putative protein (0.1)
PC4	3	4	7	8	Putative protein (0.173)
PC5	1	6	9	14	
PC6	0	0	0	1	
Total (PCs)	4	14	21	32	

*Note:* A gene was considered a candidate if the QTL is located within it or within 2000 bp upstream from its start codon. Genes with QTLs located within their exon are marked with an asterisk (*).

### 3.3 Colony‐Level GWAS

The colony‐level GWAS identified three QTLs that were found to be associated with 5Me‐C25, two of which had a *q*‐value < 0.05 (Figure 2; Table 2). These QTLs were located on different chromosomes from the loci identified in the individuals‐level analysis. Two additional QTLs were found to be associated with two other CHCs. There are 4, 5, 16, and 30 QTLs that had a *q*‐value < 5%, 10%, 20%, and 30% that were found to be associated with CHCs; of these, six were gene hits and two of the genes had a QTL in an exon. Here again, we conducted a GWAS of the first six PCs, which identified only four QTLs with a *q*‐value < 30%, all associated with PC4. We note that for the lowest significance threshold, 30% of the associations are expected to be false positives, so approximately 21 out of 30 putative QTLs are expected to be true positives.

**TABLE 2:** Candidate loci and genes that were found to be associated with CHCs in the colony‐level GWAS.

Trait	*q*‐value threshold				Genes (*q*‐value)
	0.05	0.1	0.2	0.3	
5Me‐C25	2	3	6	7	Sterile alpha and TIR motif‐containing protein 1 (0.142)
11 + 13Me‐C27	1	1	1	3	Aminopeptidase N (0.276*)
11,15DiMe‐C27	1	1	1	4	Protein TANC2 (0.298)
C28	—	—	—	7	Progestin and adipoQ receptor family member 4 (0.227*)
					Protein gooseberry‐neuro (0.261)
11 + 13Me‐C29	—	—	7	8	Claudin tight junction (0.233)
3Me‐C29	—	—	1	1	
Total (CHCs)	4	5	16	30	
PC4	0	0	0	4	
Total (PCs)	0	0	0	4	

*Note:* A gene was considered a candidate if the QTL is located within it or within 2000 bp upstream from its start codon. Genes with QTLs located within their exon are marked with an asterisk (*).

Altogether, 99 QTLs were found to be associated with CHCs with *q*‐value < 0.3, 69 in the individual‐level analysis and 30 in the colony‐level analysis. The PC‐wise analyses identified 32 and 4 loci associated with the first six PCs of the individual‐ and colony‐level PCs, respectively. The four analyses together resulted in 135 QTLs, including 10 pairs of QTLs shared that the same genetic position, and one pair that shared the same physical position (the same SNP). The QTLs distribution across the genome was found to be significantly different than a uniform distribution in a Kolmogorov–Smirnov test (*D* = 5038.9, *p*‐value < 2.2 × 10^−16^). The QTLs were clustered in 25 clusters of 2–7 loci that were within 12 cM of each other. Eight of these clusters included QTLs from both individual‐ and colony‐level analyses (Table S2). Overall, 69 of the 135 QTLs were in clusters.

Of the gene hits, some are implicated in the metabolism of amino acids. For example, pyridoxal‐dependent decarboxylase domain‐containing protein 1 contains a QTL for 4Me + 8,12DiMe‐C28. Other QTL genes are regulatory factors such as protein phosphatase Slingshot homolog 1 for C29. Other genes code for claudin, cadherin, and kinesin, which may affect cell structures and functions.

## 4 Discussion

We conducted a GWAS of CHC profiles in the desert ant *C. niger* on both the individual and the colony level. Our chosen experimental design using whole‐genome sequencing of six individuals per colony was tailored for comprehensively assessing the genetic variants of individual ants as well as for estimating the genetic composition of the colony. We note that the alternative approach of pool sequencing may also be worthwhile. In our pilot study, we estimated that the use of pools would have provided a similar statistical power for the colony‐level GWAS at a lower cost (Inbar et al. 2020). Our chemical analysis using extracts from the cuticle of an individual thorax was designed to sample the external CHC profile. We note that it may also be of interest to analyze internal samples, especially from the postpharyngeal gland that is implicated in CHC transfer among nestmates (Bagnères and Morgan 1991).

We detected a total of 99 loci associated with CHCs across the genome, and an additional 36 which were associated with the PCs of the CHCs. These results show that the genomic architecture of nestmate recognition cues is highly polygenic. We found one or more putative QTLs for 18 of the 34 CHCs (*q*‐value < 0.3)—14 in the individual‐level analysis and 6 in the colony‐level analysis. Two CHCs appeared in the results of both analyses (5Me‐C25 and 11 + 13Me‐C27). For the remaining 16 CHCs, their QTLs may have been missed due to limitations in the analysis itself, such as chemical measurement or sequencing errors, suboptimal model specification, or inaccurate kinship inference, or because the genetic contribution to their quantitative variation was too small to be detected given the limited statistical power of our GWAS with the available sample size. Notably, based on our pilot study, we estimate statistical power to be limited, ranging from approximately 64% to as low as 37% or below (see Section 2). The GWAS results for the PC axes further support this interpretation: no loci were associated with PC1, and only three with PC2. Of the 36 loci found to be associated with CHC PCs, 26 were linked to PC4 and PC5, which together explain less than 13% of the total variance in both individual‐ and colony‐level CHC profiles. We interpret this result as indicating that the first PCs, which represent the most significant axes of phenotypic variation, are the result of some environmental effects. These environmental effects are stronger than the genetic effects observed in PC4 and PC5. Nevertheless, this is the first report of QTLs for CHCs in any ant species.

While the individual‐ and the colony‐level analyses generally identified different loci as being associated with different CHCs, oftentimes QTLs of different CHCs were found close by in the genome. It has been shown that some chromosomal regions form distinct compartments in regard to their functionality and co‐regulation (Goetze et al. 2007; Matharu and Ahanger 2015). We hypothesize that the genomic clusters identified may be topological or functional domains that are related to the synthesis of CHCs. Insects synthesize hydrocarbons by elongating fatty acyl‐CoA precursors to produce long chain fatty acids that are then converted to hydrocarbons (Blomquist and Bagnères 2010). The elongation reaction is responsible for regulating hydrocarbon lengths (Blomquist and Vogt 2003) and thereby determining the variation in quantities of various CHCs with a range of chain lengths. Another source of variation is the addition of methyl branches in different positions during the synthesis of the fatty acids. Studies from various insect species identified a range of additional enzymes involved in subsequent steps of CHC synthesis, from fatty acid desaturases to decarboxylases (Blomquist and Bagnères 2010). Complex mechanisms may be involved in the trafficking of CHCs and their secretion to the cuticle (Blomquist and Bagnères 2010), including the likely involvement of the postpharyngeal gland (Bagnères and Morgan 1991). CHCs are transported through the hemolymph bound to lipophorin, and then deposited to the cuticle. Considerable selectivity was demonstrated at this stage in some species, such as the termite *Zootermopsis nevadensis* , where the hemolymph hydrocarbon profile was similar across castes, but the cuticular profiles of different castes were quantitatively different in terms of the proportions of the hydrocarbons (Sevala et al. 2002). The candidate QTLs that we discovered may be related to such biosynthetic and trafficking mechanisms, perhaps as polymorphic regulatory factors. We also note that most of the QTLs do not affect the protein coding sequences of genes. We suggest that these SNPs affect regulatory elements that may modulate transcription or epigenetic mechanisms that regulate gene expression. Further study of the candidate loci is needed in order to examine their relation to CHC synthesis. Future functional genomic studies (e.g., RNA sequencing or bisulfite sequencing) could provide additional insights into the molecular mechanisms responsible for regulatory effects of CHC QTLs.

The ability to discriminate between nestmates and non‐nestmates depends on the differences between their CHC profiles (Lahav et al. 1999; Wagner et al. 2000; Drescher et al. 2010; Foitzik et al. 2007; Blight et al. 2012). We may expect that the more distinct their colony CHC profile is, the easier it would be for nestmates to identify each other. Colonies with rare genotypes in loci that regulate CHCs would therefore be favored by natural selection over colonies with common genotypes. Rare alleles would be selected for so long as they are rare, thus maintaining and increasing the polymorphism in the population. Therefore, we may predict balancing (frequency‐dependent) selection on genes that regulate CHCs, in a similar fashion as in other recognition systems for which polymorphism is advantageous for the discrimination of self vs. non‐self, such as the MHC immunity system in mammals (Hedrick 1998; Aguilar et al. 2004) and the self‐incompatibility system of plants (Glémin et al. 2005). This leads us to predict that CHC variation will increase in evolution through the generation of polymorphic genetic loci that affect CHC synthesis and/or their secretion on the cuticle, i.e., CHC QTLs.

Despite this prediction for the evolution of genetic polymorphism in nestmate recognition cues, environmental factors may have greater contribution than genetic factors to the phenotypic variation. In our analysis, only seven QTLs for four of the 34 CHCs were detected with a *q*‐value < 0.05; two CHCs in the individual‐ and three in the colony‐level analysis (QTLs for CHC 5Me‐C25 were detected in both analyses). Diet was shown to influence the cuticular chemical profiles in the polygyne and polydomous colonies of *Formica aquilonia* ants, where nestmates are not necessarily related (Sorvari et al. 2008). In comparison, the colonies of *C. niger* used in this study are monogyne (Brodetzki et al. 2019), and so nestmates are always related (at least 25% relatedness). Environmental odors also appear to determine aggression levels between lab‐reared and field‐collected workers of monogyne *Solenopsis invicta* colonies (Obin 1986) and similarly between lab‐reared and field‐collected polygyne *Rhytidoponera confusa* workers (M. W. Crosland 1989). Considering the limited results from our GWAS, it appears that genetic factors have a lesser role than environmental factors in our study system. Indeed, the level of statistical significance of our results indicates that the percent explained variability (PEV) of CHC QTLs in our study population is less than the 23% PEV that we simulated in our pilot study (see Section 2). While previous studies employed various approaches to reveal evidence for genetic effects on ant CHCs, our GWAS is the first to our knowledge that is able to show that the magnitude of genetic effects is quantitatively smaller than that of environmental effects.

Nevertheless, our analyses suggest that polymorphism in multiple genes contributes to CHC variation. The statistical power obtained from the sample size of 244 ants from 45 colonies limited our confidence in specific QTLs, yet we can conclude from the results that tens of QTLs affect CHCs. This observation is in line with studies in model insect species such as *Drosophila*, where larger sample sizes and more powerful experimental approaches are available. For example, a GWAS of CHCs conducted using 169 inbred lines from the *D. melanogaster* Genetic Reference Panel (DGRP) identified QTLs in 305 and 173 genes in females and males, respectively (Dembeck et al. 2015). Also in *Drosophila*, it is likely that the sample size limits the statistical power of GWAS. The numbers of QTLs reported are likely underestimates because QTLs with smaller effect sizes are more difficult to detect. Therefore, we may conclude that CHCs have a complex polygenic genomic architecture, which is in line with their being complex quantitative traits.

The Gestalt model suggests that social interaction between colony members has an important role in determining colony identity. Social interactions have substantial impacts on many other phenotypes in insect societies. For example, when *Camponotus floridanus* workers were fed with juvenile hormone, an important regulator of growth and development in insects, the larvae they fed through trophallaxis were twice as likely to become larger workers (LeBoeuf et al. 2019). In termites, social interactions of allogrooming and trophallaxis appear to contribute in determining the caste of developing brood (Watanabe et al. 2014). The physical transfer and mixing of CHCs between nestmates may be a major factor shaping their chemical identity. The Gestalt model motivated us to conduct the colony‐level GWAS. We formulated the colony‐level genotypes and phenotypes as the arithmetic means of the individual genotypes and phenotypes. We note that we used a random sample of six workers that represents the full population of the nest, including all sizes of nurses and foragers. When considering genotypes, the arithmetic mean gives the allele frequency in our sample of six workers, which is an estimate of the allele frequency in the colony, assuming that this is a representative sample of the worker population. The choice of an arithmetic mean of the phenotypes is less obvious. We chose an arithmetic mean because it is the simplest, most natural summary statistic. However, this choice implies the assumptions that (Hamilton 1964) each worker contributes equally to the colony odor and that (Hamilton 1987) these contributions are additive. Both assumptions may be incorrect. Obviously, larger workers could be expected to contribute more CHCs than smaller ones. Also, nurses and foragers may contribute differently because they may produce different CHC profiles (e.g., Wagner et al. 1998, 2001) and because social interactions are structured by task group (e.g., Mersch et al. 2013). Ideally, we should have accounted for such factors in our model. However, it is not known which factors affect the mixing of CHCs and to what extent. Therefore, we elected the simplest model of arithmetic mean. The colony‐level analysis resulted in fewer QTLs identified in comparison to the individual‐level analysis, but in general with greater statistical significance, indicating more confidence in the colony‐level model. This result may be interpreted as supporting the Gestalt model. Alternatively, it may be that the averaging of colonies' genotypes and phenotypes helps in eliminating noise introduced in the chemical analysis and sequencing/genotype errors, and so results in a more robust outcome. Additional studies including behavioral analysis and manipulation may provide further insight into the significance of the Gestalt model and determine the exact nature of social effects on CHC profiles. Whichever is the case, our colony‐level analysis was successful in revealing putative QTLs, indicating that this approach is appropriate for CHCs. Whether the Gestalt effect is present or not, genomic mapping of CHCs should be done at the colony level due to the shared genetics and environment of nestmates.

## Author Contributions

**Pnina Cohen:** conceptualization (equal), data curation (equal), formal analysis (lead), investigation (equal), methodology (equal), software (equal), visualization (lead), writing – original draft (lead), writing – review and editing (equal). **Shani Inbar:** conceptualization (equal), data curation (lead), formal analysis (equal), investigation (equal), methodology (equal), resources (lead). **Eyal Privman:** conceptualization (lead), funding acquisition (lead), investigation (equal), methodology (equal), project administration (lead), resources (equal), software (equal), supervision (lead), writing – review and editing (equal).

## Conflicts of Interest

The authors declare no conflicts of interest.

## Supporting information


**Data S1:** ece372726‐sup‐0001‐DataS1.zip.
**Table S3:** Relative quantities of CHCs for each sample.

## Data Availability

Genomic sequencing data were submitted to the NCBI SRA database under BioProject accession PRJNA1168157. Chemical data are available as Supporting Information to this manuscript. Scripts were submitted to the Zenodo repository (https://doi.org/10.5281/zenodo.16729334).
