# Supplementary material for: Genomic Architecture of Nestmate Recognition Cues in the Desert Ant
Source: Ecol Evol. 2025 Dec 23;15(12):e72726. doi: 10.1002/ece3.72726 (PMC12723442; doi:10.1002/ece3.72726)
Supplement: Supplementary file 1 — Data S1: ece372726‐sup‐0001‐DataS1.zip. Table S3: Relative quantities of CHCs for each sample. [file ECE3-15-e72726-s001.zip › ece372726-sup-0001-supinfo.docx]

# Supplementary materials


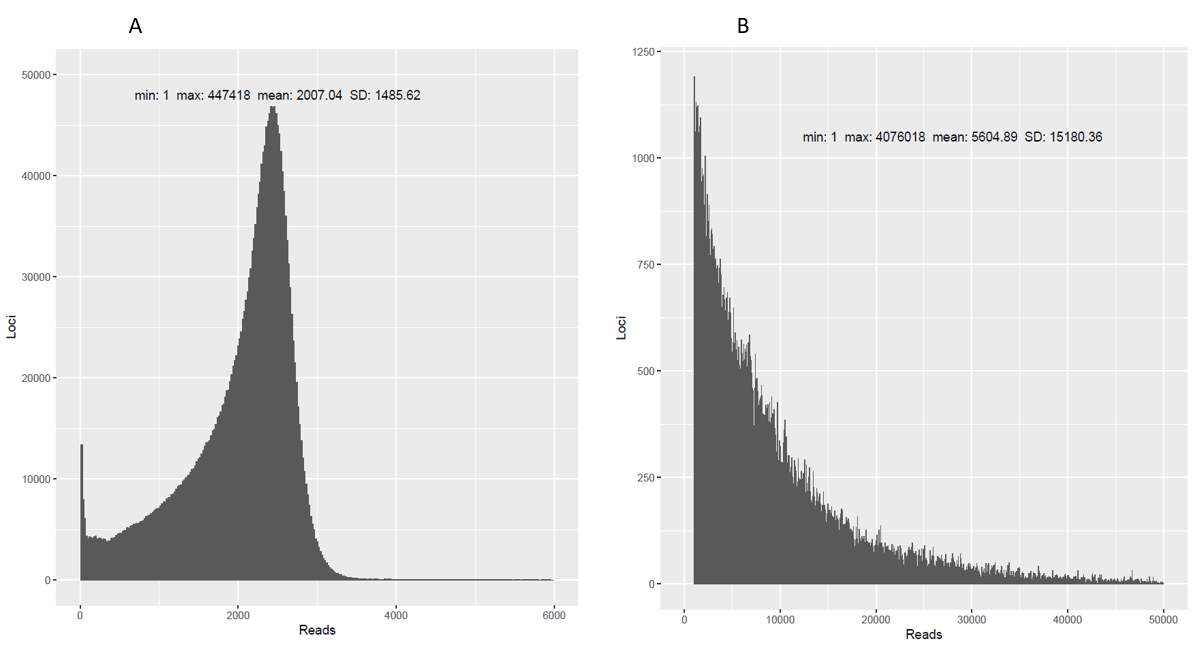


**Figure S1.** Distribution and summary statistics of the number of reads across unfiltered loci. (A) 276 fully sequenced samples from 47 colonies sampled in Betzet beach in northern Israel. (B) 47 RAD-sequenced samples of 47 colonies from which the fully sequenced samples also originate.

**Figure S2**. Example chromatogram of cuticular wash of *Cataglyphis* *niger* from the Betzet Beach population. Below are the spectra of 2 of the monomethyl hydrocarbons. (A) 3-methylpentacosane, characterised by fragments at m/z 56/337. (B) a mixture of coeluted 13- and 15-methylnonacosane. The fragments 196/252 represent branching on the 13 carbon and the fragment at m/z 224 (the molecule broke to two equal fragments) represents the branching at carbon 15.


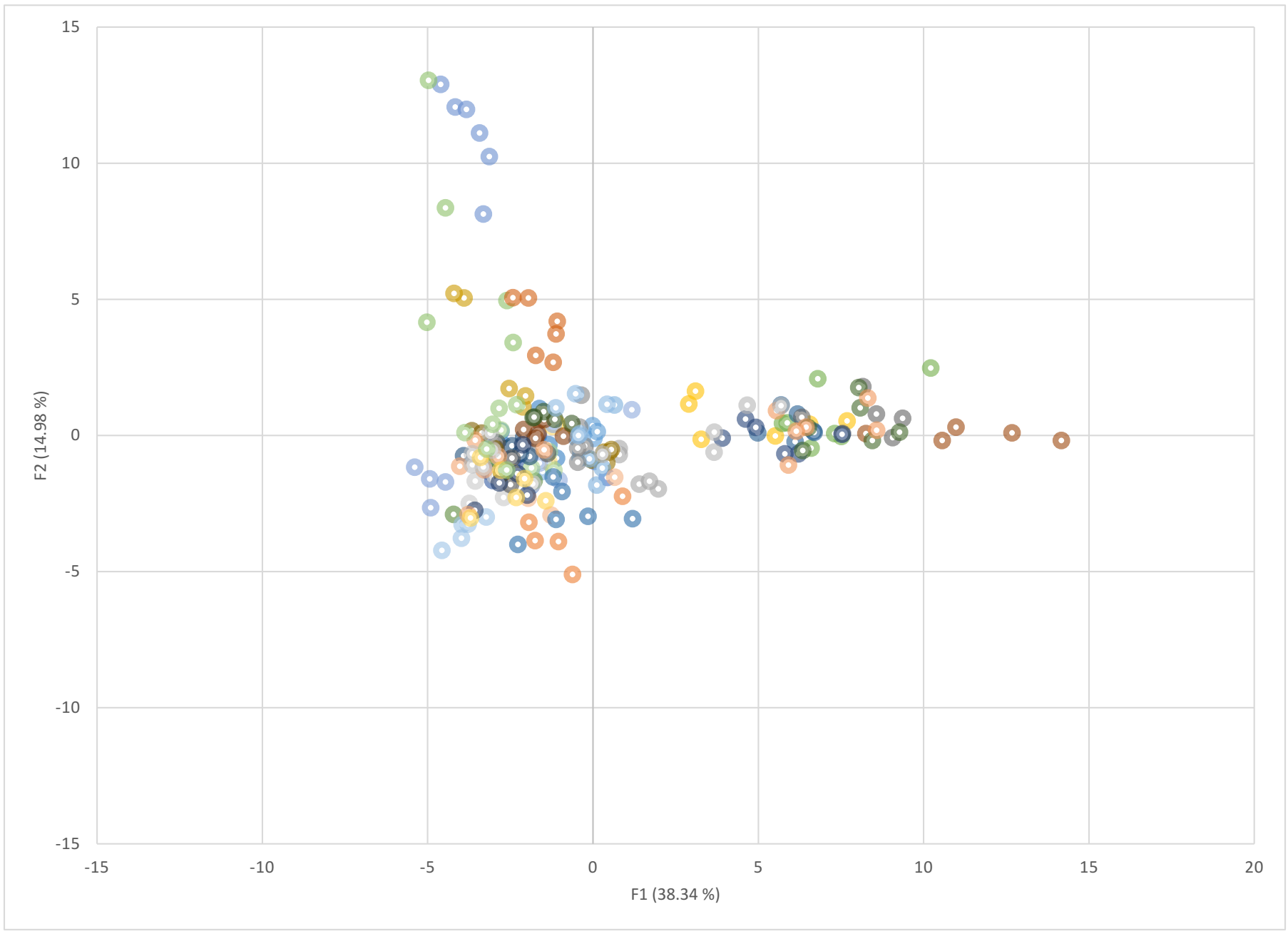


**Figure S3**. Linear discriminant analysis (DA) of the chemical signature of all colonies examined in this study. Samples from different nests are marked by different colors. Wilks' Lambda test (Rao's approximation) indicate a statistical difference between colonies (p-value<0.0001).


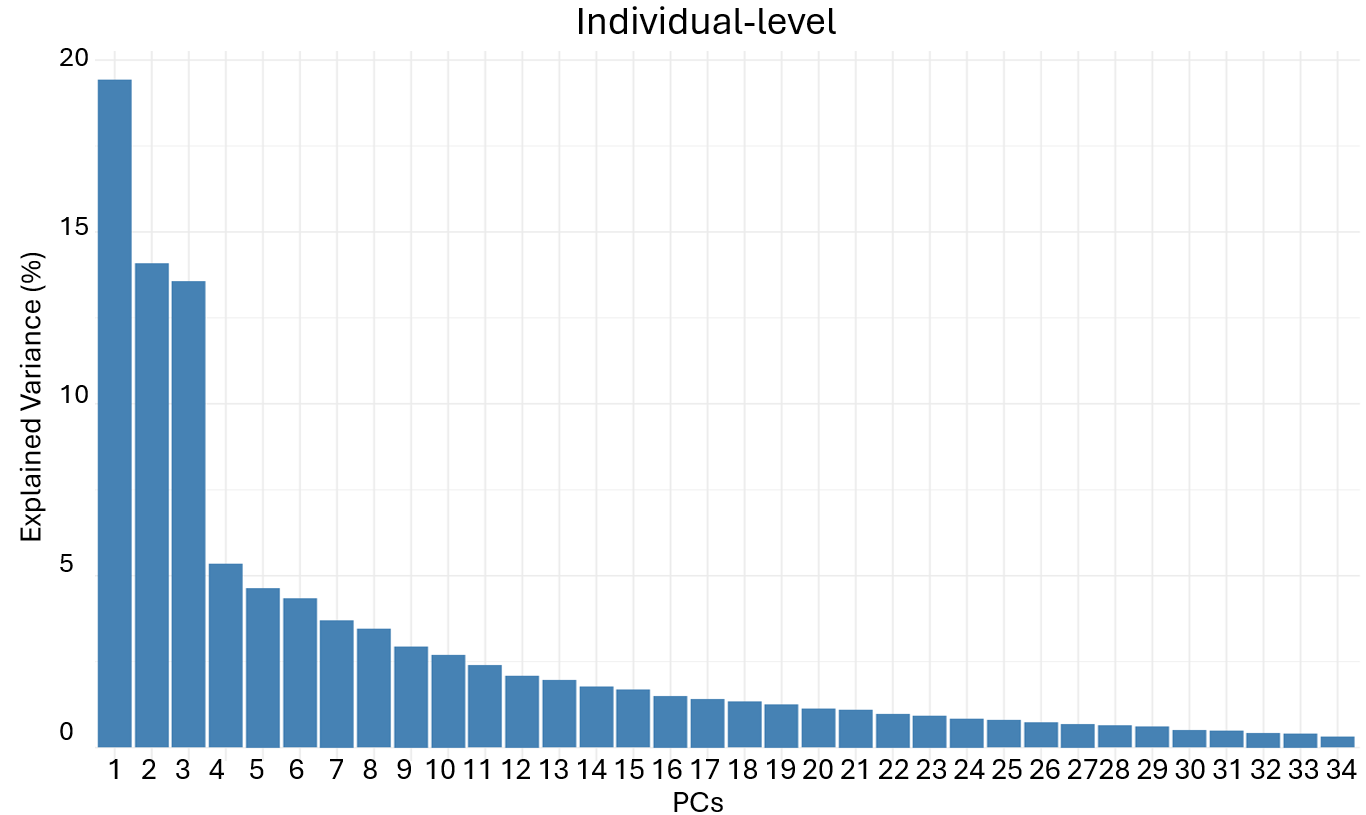


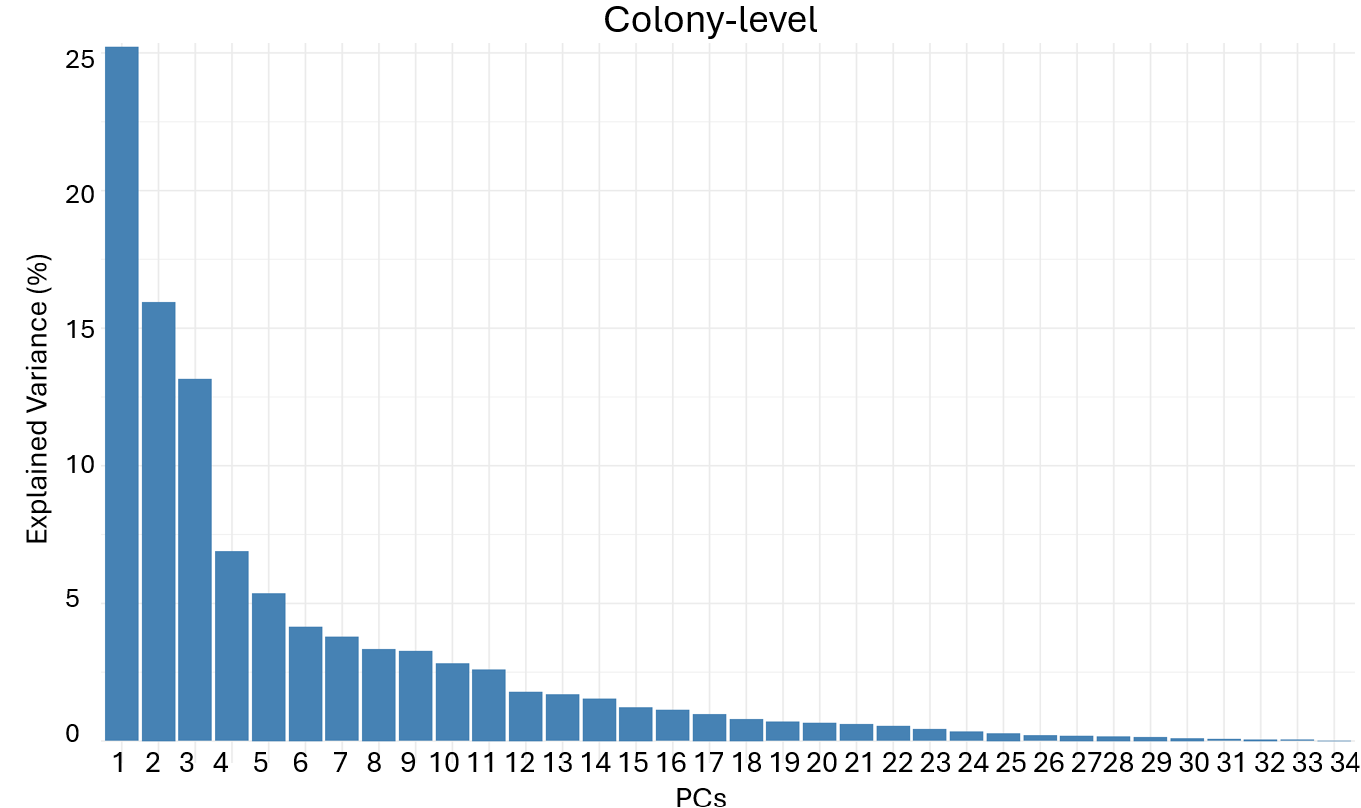


Figure S4. Explained variance by each PC in the individual and colony-level trait PCA


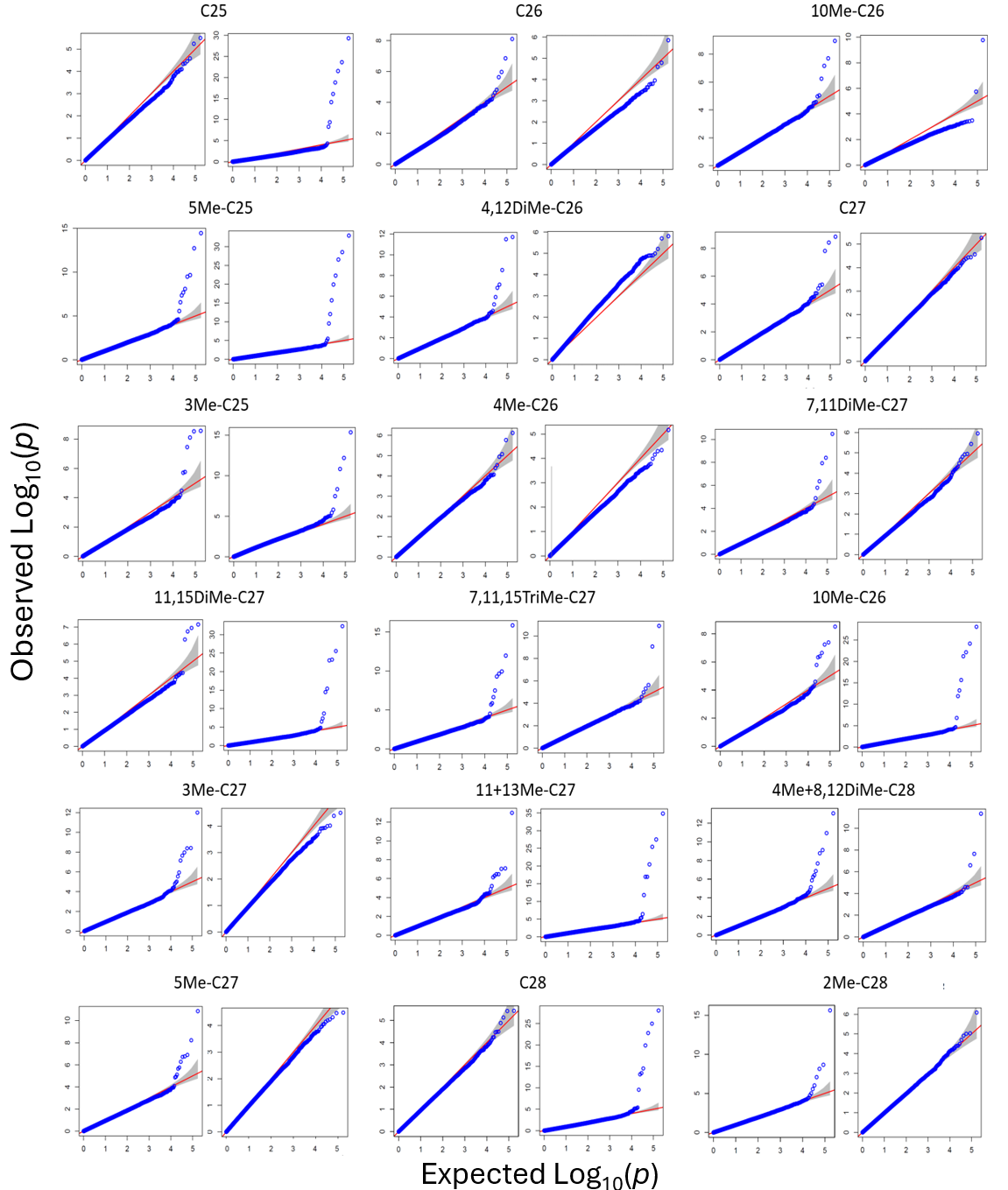


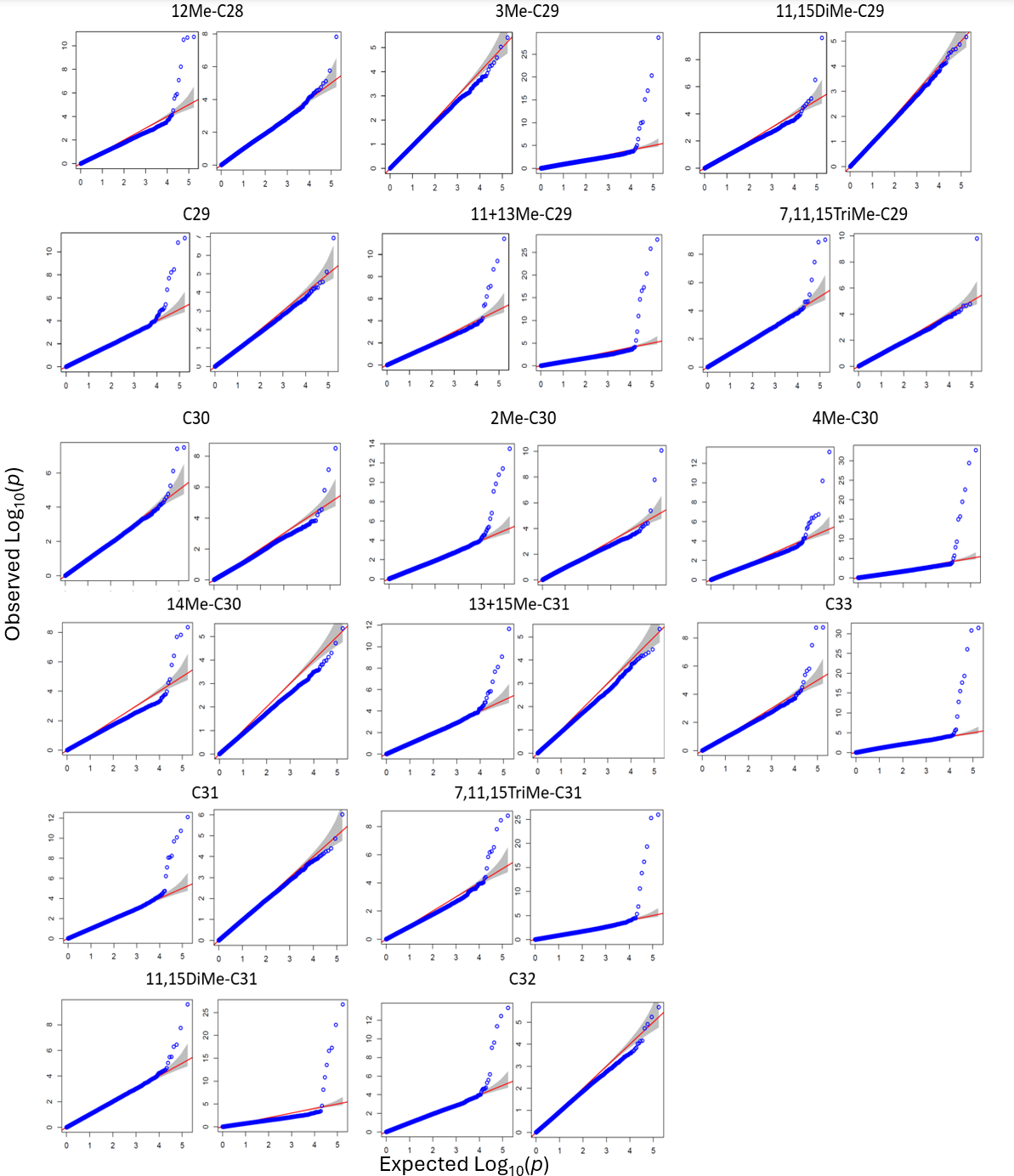


**Figure S5.** Assessment of how well the model used in the individual-level (left) and colony-level (right) analyses accounts for familial relatedness and population structure for each of the CHC traits. The negative logarithms of the loci *p-*values S are plotted against their expected value under the null hypothesis of no association with the trait.


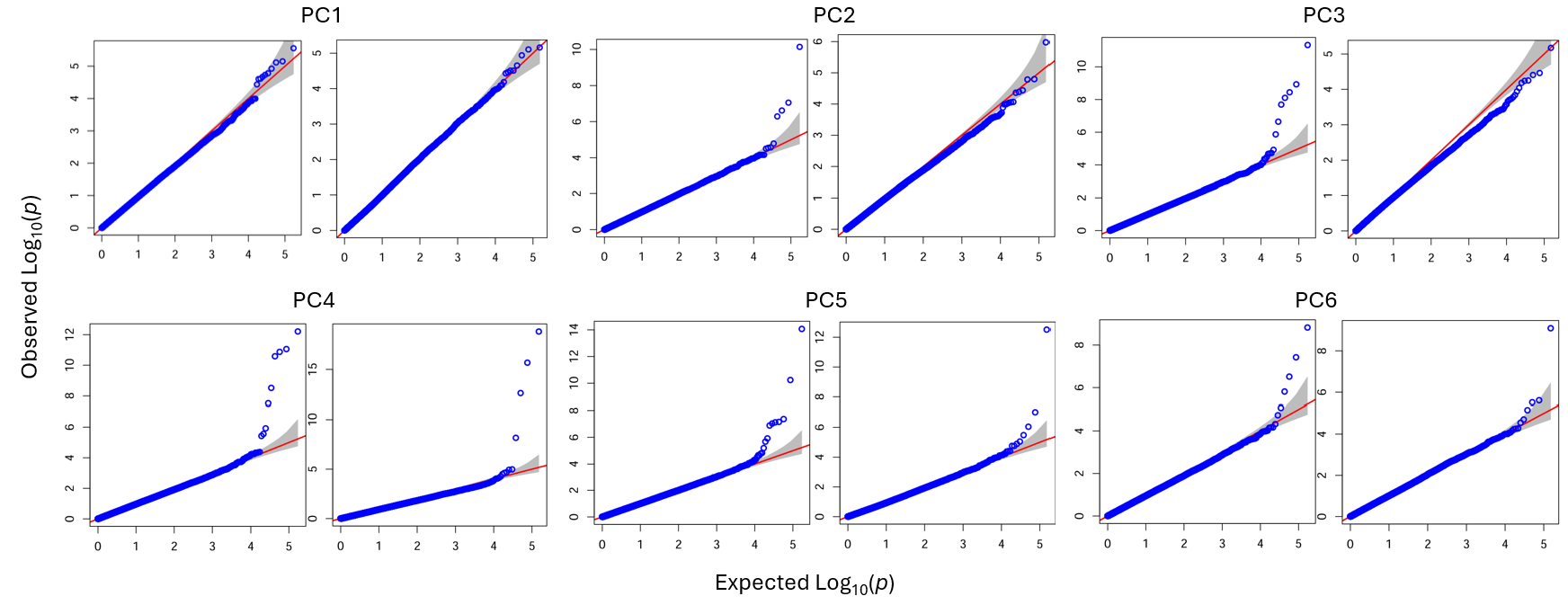


**Figure S6.** Assessment of how well the model used in the individual- (left) and colony-level (right) analyses accounts for familial relatedness and population structure for the first PCs of the CHC traits. The negative logarithms of the loci *p-*values S are plotted against their expected value under the null hypothesis of no association with the trait.

**Figure S7.** Principle Component Analysis (PCA) of genotype data from 244 individuals of 45 colonies colored by colony ID. Note that colonies were numbered from north to south along the transect.

A

B****

**Figure S8.** Kinship matrices based on the total length of shared IBD haplotypes between 244 individuals of 45 colonies (A) and 47 individuals of 47 colonies (B). For visibility, only colony numbers are presented. The heatmap coloring is based on the total number of bases in shared haplotypes between each pair of samples. Higher values are stronger red.

**Table S1**. Outlayer removals and normalization methods of the phenotype on the individual and colony level.

| **Trait** | **Individual-level outlayers** | **Individual-level normalization** | **Colony-level outlayers** | **Colony-level normalization** |
| --- | --- | --- | --- | --- |
| C25 | 17 | Yeo-Johnson | 0 | Log_b(x + a) |
| 5Me-C25 | 44 | Order norm | 9 | Log_b(x + a) |
| 3Me-C25 | 22 | Log_b(x + a) | 4 | Box Cox |
| C26 | 23 | sqrt(x + a) | 2 | Box Cox |
| 10Me-C26 | 13 | Log_b(x + a) | 2 | sqrt(x + a) |
| 4Me-C26 | 20 | Order norm | 3 | Log_b(x + a) |
| 4,12DiMe-C26 | 12 | asinh(x) | 1 | sqrt(x + a) |
| C27 | 11 | Order norm | 2 | Log_b(x + a) |
| 5Me-C257 | 5 | asinh(x) | 0 | sqrt(x + a) |
| 11+13Me-C27 | 13 | Order norm | 2 | Log_b(x + a) |
| 11,15DiMe-C27 | 37 | Order norm | 9 | Log_b(x + a) |
| 7,11DiMe-C27 | 2 | Yeo-Johnson | 0 | asinh(x) |
| 3Me-C27 | 16 | Box Cox | 1 | Box Cox |
| 7,11,15TriMe-C27 | 21 | exp(x) | 4 | asinh(x) |
| C28 | 37 | Order norm | 4 | sqrt(x + a) |
| 12Me-C28 | 13 | Order norm | 0 | asinh(x) |
| 4,12DiMe-C28 | 1 | Box Cox | 0 | sqrt(x + a) |
| 4Me+8,12DiMe-C28 | 16 | sqrt(x + a) | 0 | Box Cox |
| 2Me-C28 | 23 | Order norm | 2 | sqrt(x + a) |
| C29 | 14 | Box Cox | 1 | sqrt(x + a) |
| 11+13Me-C29 | 13 | Box Cox | 1 | Box Cox |
| 11,15DiMe-C29 | 17 | Yeo-Johnson | 2 | Log_b(x + a) |
| 3Me-C29 | 34 | Order norm | 3 | sqrt(x + a) |
| 7,11,15TriMe-C29 | 5 | Box Cox | 3 | sqrt(x + a) |
| C30 | 26 | Yeo-Johnson | 4 | asinh(x) |
| 14Me-C30 | 15 | Box Cox | 2 | asinh(x) |
| 4Me-C30 | 16 | Order norm | 0 | Log_b(x + a) |
| 2Me-C30 | 14 | asinh(x) | 4 | Log_b(x + a) |
| C31 | 14 | Log_b(x + a) | 3 | Box Cox |
| 13+15Me-C31 | 11 | exp(x) | 4 | sqrt(x + a) |
| 11,15DiMe-C31 | 11 | Box Cox | 0 | Log_b(x + a) |
| 7,11,15TriMe-C31 | 4 | Box Cox | 0 | asinh(x) |
| C32 | 13 | asinh(x) | 4 | asinh(x) |
| C33 | 8 | asinh(x) | 0 | sqrt(x + a) |

**Table S2. The mapping of significant loci (*q*-value < 0.3) on the genome. Trais-wise,** individual-level GWAS results are in blue and colony-level GWAS results are in black**.** PC-wise, individual-level GWAS results are in light blue and colony-level GWAS results are in gray. Loci that are mapped to the same or close genetic position (within 12cM) are marked with yellow or orange backgrounds. Both genetic and physical positioning are shown.

| **Chr** | **Genetic pos** | **Scaffold** | **Position** | **CHC** | **q-value** |
| --- | --- | --- | --- | --- | --- |
| 1 | 0 | scaffold17\|size760598 | 515848 | 4,12DiMe-C26 | 0.279715055 |
| 1 | 2.87 | scaffold35\|size591243 | 473891 | 4,12DiMe-C26 | 0.11352072 |
| 1 | 9.66 | scaffold296\|size210905 | 44019 | 4,12DiMe-C26 | 0.279715055 |
| 1 | 24.73 | scaffold265\|size229190 | 31670 | PC5 | 0.098600854 |
| 1 | 24.73 | scaffold265\|size229190 | 145912 | PC4 | 0.187309189 |
| 1 | 34.15 | scaffold578\|size105271 | 38187 | 7,11,15TriMe-C27 | 0.168010666 |
| 1 | 88.15 | scaffold916\|size58012 | 4774 | C31 | 0.118061549 |
| 1 | 88.15 | scaffold293\|size211391 | 198932 | C33 | 0.281531387 |
| 2 | 65.4 | scaffold636\|size94593 | 82725 | 3Me-C25 | 0.281531387 |
| 2 | 107.49 | scaffold222\|size263138 | 94041 | PC4 | 0.262551622 |
| 3 | 85.67 | scaffold320\|size199217 | 36954 | PC5 | 0.018163315 |
| 3 | 85.67 | scaffold320\|size199217 | 33276 | 11+13Me-C27 | 0.259138689 |
| 3 | 87.52 | scaffold1113\|size42492 | 15042 | 4Me+8,12DiMe-C28 | 0.168010666 |
| 4 | 1.88 | scaffold1163\|size40329 | 39019 | 4Me+8,12DiMe-C28 | 0.201612799 |
| 4 | 16.6 | scaffold558\|size111151 | 58293 | 11+13Me-C29 | 0.145481369 |
| 4 | 39.56 | scaffold77\|size466230 | 39015 | 4,12DiMe-C26 | 0.23006866 |
| 4 | 39.56 | scaffold551\|size113931 | 79453 | C31 | 0.144009142 |
| 4 | 83.57 | scaffold326\|size196711 | 73375 | 11+13Me-C27 | 0.009092586 |
| 4 | 143.72 | scaffold220\|size265453 | 70637 | C32 | 0.163469837 |
| 4 | 181.71 | scaffold734\|size79827 | 17289 | 7,11,15TriMe-C27 | 0.23793943 |
| 4 | 203.11 | scaffold74\|size472645 | 164044 | PC3 | 0.09989823 |
| 4 | 203.11 | scaffold74\|size472645 | 164044 | C31 | 0.118061549 |
| 5 | 96.41 | scaffold146\|size337763 | 96934 | PC5 | 0.25580002 |
| 5 | 131.68 | scaffold35\|size591243 | 43412 | 5Me-C25 | 0.106385132 |
| 5 | 155.42 | scaffold467\|size138454 | 17129 | PC4 | 0.02421775 |
| 6 | 70.38 | scaffold336\|size190304 | 25911 | C29m3 | 0.181851711 |
| 6 | 90.66 | scaffold737\|size79726 | 15861 | C28 | 0.251561534 |
| 7 | 13.82 | scaffold183\|size295869 | 189137 | PC4 | 0.024217753 |
| 7 | 52.75 | scaffold107\|size407829 | 186705 | C33 | 0.281531387 |
| 7 | 56.43 | scaffold859\|size63718 | 3120 | 7,11,15TriMe-C27 | 0.168010666 |
| 7 | 57.35 | scaffold19\|size722566 | 429439 | C31 | 0.144009142 |
| 7 | 62.08 | scaffold279\|size219011 | 173386 | 11,15DiMe-C27 | 0.018185171 |
| 7 | 62.08 | scaffold352\|size184572 | 137082 | PC5 | 0.14303610 |
| 7 | 64.02 | scaffold59\|size504363 | 457249 | PC5 | 0.098600854 |
| 7 | 101.52 | scaffold30\|size631625 | 144566 | C28 | 0.275375449 |
| 7 | 104.36 | scaffold85\|size450463 | 361438 | 11+13Me-C29 | 0.175789988 |
| 7 | 111.1 | scaffold28\|size641708 | 145558 | 4Me+8,12DiMe-C28 | 0.21114854 |
| 7 | 135.68 | scaffold404\|size159223 | 63505 | 3Me-C25 | 0.281531387 |
| 8 | 126.41 | scaffold194\|size285062 | 81296 | 11+13Me-C29 | 0.175789988 |
| 8 | 137.97 | scaffold618\|size98603 | 6938 | C31 | 0.118061549 |
| 8 | 208.22 | scaffold658\|size91109 | 44118 | 11,15DiMe-C27 | 0.297782177 |
| 8 | 154.22 | scaffold242\|size245525 | 114890 | PC2 | 0.224771026 |
| 8 | 222.43 | scaffold740\|size79674 | 67018 | C32 | 0.096871015 |
| 8 | 227.33 | scaffold654\|size92139 | 4110 | PC4 | 0.295370576 |
| 8 | 259.2 | scaffold20\|size721085 | 484650 | C29 | 0.28516405 |
| 8 | 309.3 | scaffold751\|size78167 | 6597 | 7,11,15TriMe-C29 | 0.287585825 |
| 9 | 45.61 | scaffold54\|size511285 | 140799 | 5Me-C25 | 0.009092586 |
| 9 | 53.53 | scaffold244\|size244552 | 117700 | PC4 | 0.262551623 |
| 9 | 85.74 | scaffold235\|size251712 | 52728 | C32 | 0.090816576 |
| 9 | 90.85 | scaffold260\|size234419 | 201342 | PC5 | 0.255800023 |
| 9 | 119.36 | scaffold219\|size265940 | 180069 | 7,11,15TriMe-C27 | 0.168010666 |
| 9 | 137.13 | scaffold97\|size425009 | 229095 | PC3 | 0.099898234 |
| 9 | 137.13 | scaffold311\|size203550 | 120617 | C29 | 0.186173981 |
| 9 | 142.85 | scaffold505\|size124814 | 43537 | 12Me-C28 | 0.093843796 |
| 9 | 142.85 | scaffold66\|size491290 | 166504 | 4Me+8,12DiMe-C28 | 0.276082392 |
| 10 | 46.63 | scaffold274\|size221563 | 83187 | PC4 | 0.172551495 |
| 10 | 105.07 | scaffold306\|size205911 | 65549 | 12Me-C28 | 0.093843796 |
| 10 | 131.82 | scaffold75\|size469975 | 227297 | PC5 | 0.086275748 |
| 10 | 132.88 | scaffold75\|size469975 | 364802 | 4Me+8,12DiMe-C28 | 0.21114854 |
| 11 | 2.02 | scaffold146\|size337763 | 226678 | 7,11,15TriMe-C27 | 0.271314522 |
| 11 | 17.44 | scaffold291\|size213446 | 9721 | 4Me-C30 | 0.099898234 |
| 11 | 44.83 | scaffold22\|size714790 | 90604 | 11+13Me-C27 | 0.275808429 |
| 11 | 111.65 | scaffold67\|size489733 | 28054 | C31 | 0.189579603 |
| 11 | 153.29 | scaffold622\|size97615 | 36744 | 3Me-C25 | 0.292883459 |
| 11 | 172.12 | scaffold105\|size412506 | 116781 | PC5 | 0.098600854 |
| 11 | 312.17 | scaffold508\|size123518 | 117137 | 12Me-C28 | 0.093843796 |
| 12 | 0 | scaffold730\|size80008 | 50311 | 2Me-C30 | 0.240663927 |
| 12 | 67.25 | scaffold71\|size482159 | 21063 | 4Me+8,12DiMe-C28 | 0.21114854 |
| 12 | 77.84 | scaffold71\|size482159 | 252805 | 4Me+8,12DiMe-C28 | 0.215941637 |
| 12 | 87.97 | scaffold593\|size103847 | 95748 | 11+13Me-C29 | 0.19614006 |
| 12 | 94.99 | scaffold65\|size491780 | 101529 | 7,11,15TriMe-C27 | 0.271314522 |
| 12 | 115.45 | scaffold209\|size272198 | 146941 | 5Me-C25 | 0.045408288 |
| 12 | 141.58 | scaffold470\|size137511 | 24197 | C31 | 0.226032368 |
| 14 | 40.37 | scaffold44\|size549762 | 519588 | 11+13Me-C29 | 0.175789988 |
| 14 | 48.21 | scaffold581\|size104792 | 64268 | 5Me-C25 | 0.207830527 |
| 14 | 50.17 | scaffold125\|size365959 | 224995 | 7,11,15TriMe-C29 | 0.254286414 |
| 14 | 92.55 | scaffold204\|size276989 | 165280 | 4Me+8,12DiMe-C28 | 0.072653261 |
| 14 | 95.38 | scaffold144\|size340398 | 186437 | 5Me-C25 | 0.141844335 |
| 14 | 107.79 | scaffold3\|size1157121 | 1010964 | 2Me-C30 | 0.265638486 |
| 14 | 127.94 | scaffold130\|size360227 | 149410 | 4Me+8,12DiMe-C28 | 0.168010666 |
| 15 | 52.68 | scaffold72\|size474807 | 247511 | 4,12DiMe-C26 | 0.11352072 |
| 15 | 90.15 | scaffold124\|size367063 | 14584 | 5Me-C25 | 0.093086991 |
| 15 | 94.21 | scaffold99\|size421651 | 410341 | 5Me-C25 | 0.027244973 |
| 15 | 163.06 | scaffold8\|size894206 | 397978 | PC2 | 0.108979892 |
| 15 | 163.06 | scaffold8\|size894206 | 450268 | PC5 | 0.098600854 |
| 15 | 174.71 | scaffold212\|size271006 | 160026 | 11+13Me-C27 | 0.172551495 |
| 16 | 10.01 | scaffold676\|size88242 | 44336 | PC5 | 0.258178553 |
| 16 | 10.01 | scaffold676\|size88242 | 44338 | PC5 | 0.258178553 |
| 16 | 40.47 | scaffold1\|size1555347 | 1092532 | 11,15DiMe-C27 | 0.297782177 |
| 16 | 42.28 | scaffold330\|size193721 | 88898 | PC2 | 0.224771026 |
| 17 | 55.6 | scaffold299\|size209978 | 118401 | C31 | 0.118061549 |
| 17 | 68.45 | scaffold29\|size639771 | 423039 | 5Me-C25 | 0.072740685 |
| 17 | 147.15 | scaffold608\|size100611 | 57366 | 7,11,15TriMe-C27 | 0.018163315 |
| 18 | 74.77 | scaffold153\|size330014 | 258009 | 12Me-C28 | 0.220230198 |
| 18 | 89.1 | scaffold304\|size207121 | 9232 | PC4 | 0.187309189 |
| 18 | 117.35 | scaffold61\|size494520 | 99800 | 11+13Me-C29 | 0.175789988 |
| 18 | 136.63 | scaffold13\|size775888 | 175137 | 5Me-C25 | 0.141844335 |
| 18 | 148.94 | scaffold74\|size472645 | 467205 | C28 | 0.227314639 |
| 20 | 0 | scaffold1423\|size26545 | 12315 | 5Me-C25 | 0.181851711 |
| 20 | 83.75 | scaffold53\|size513719 | 234805 | 4Me+8,12DiMe-C28 | 0.108979892 |
| 20 | 110.88 | scaffold392\|size162570 | 49495 | 5Me-C25 | 0.045462928 |
| 21 | 56.5 | scaffold52\|size514423 | 369128 | 7,11,15TriMe-C29 | 0.254286414 |
| 22 | 25.72 | scaffold181\|size296900 | 135327 | PC3 | 0.072653261 |
| 22 | 85.98 | scaffold271\|size223719 | 127531 | C28 | 0.24549981 |
| 23 | 19.07 | scaffold16\|size761867 | 381681 | 3Me-C25 | 0.281531387 |
| 23 | 45.16 | scaffold180\|size296944 | 252701 | C29 | 0.28516405 |
| 23 | 121.16 | scaffold565\|size108858 | 52888 | PC4 | 0.061301189 |
| 23 | 252.96 | scaffold588\|size104489 | 7055 | C29 | 0.28516405 |
| 23 | 278.7 | scaffold583\|size104635 | 55219 | C28 | 0.275375449 |
| 23 | 305.95 | scaffold50\|size528598 | 132718 | PC3 | 0.23793943 |
| 23 | 339.08 | scaffold678\|size88160 | 68948 | PC5 | 0.098600854 |
| 23 | 346.55 | scaffold27\|size651064 | 287045 | C32 | 0.174367827 |
| 23 | 347.46 | scaffold113\|size393933 | 273321 | 2Me-C30 | 0.118061549 |
| 23 | 370.92 | scaffold232\|size254158 | 2527 | 5Me-C25 | 0.106385132 |
| 24 | 124.87 | scaffold283\|size217798 | 153213 | PC5 | 0.149342814 |
| 24 | 126.75 | scaffold29\|size639771 | 105088 | PC6 | 0.290613044 |
| 24 | 143.62 | scaffold323\|size197691 | 182834 | 2Me-C30 | 0.265638486 |
| 24 | 161.75 | scaffold4\|size1059764 | 903492 | PC3 | 0.099898233 |
| 24 | 189.98 | scaffold628\|size95885 | 66061 | C29 | 0.186173981 |
| 24 | 218.66 | scaffold9\|size875129 | 472778 | PC4 | 0.262551623 |
| 24 | 243.79 | scaffold215\|size268708 | 257542 | 11+13Me-C29 | 0.232997505 |
| 24 | 284.75 | scaffold26\|size661983 | 198368 | 5Me-C25 | 0.106385132 |
| 25 | 67.31 | scaffold6\|size960371 | 700278 | C32 | 0.090816576 |
| 25 | 84.71 | scaffold155\|size327501 | 273726 | C31 | 0.144009142 |
| 25 | 90.77 | scaffold517\|size122010 | 17255 | PC4 | 0.024217753 |
| 25 | 94.57 | scaffold19\|size722566 | 309358 | C28 | 0.261411835 |
| 26 | 21.23 | scaffold553\|size113576 | 90729 | 11,15DiMe-C27 | 0.297782177 |
| 26 | 33.21 | scaffold33\|size606826 | 197846 | 12Me-C28 | 0.292429376 |
| 26 | 35.08 | scaffold276\|size220912 | 66817 | PC2 | 0.22477102 |
| 26 | 127.17 | scaffold804\|size70630 | 40423 | 5Me-C25 | 0.093086991 |
| 26 | 127.17 | scaffold205\|size273502 | 80731 | PC5 | 0.20070463 |
| 26 | 221.23 | scaffold57\|size507111 | 381549 | C28 | 0.275375449 |
| 26 | 278.97 | scaffold303\|size207491 | 10542 | 11+13Me-C29 | 0.145481369 |
| 26 | 309.97 | scaffold8\|size894206 | 191498 | 7,11,15TriMe-C27 | 0.264881681 |
